# The latest Fontan paradox: when less is more (difficult)

**DOI:** 10.1093/europace/euag239

**Published:** 2026-09-14

**Authors:** Jeremy P Moore, Thomas Paul, Susan P Etheridge

**Affiliations:** University of California Los Angeles (UCLA) Cardiac Arrhythmia Center, UCLA Health System, Los Angeles, CA, USA; Ahmanson/UCLA Adult Congenital Heart Disease Center, Department of Medicine, Division of Cardiology, Los Angeles, CA, USA; Division of Pediatric Cardiology, Department of Pediatrics, David Geffen School of Medicine, UCLA Health System, Los Angeles, CA, USA; Department of Pediatric Cardiology, Intensive Care Medicine and Neonatology, University Medical Center, Georg-August-University Göttingen, Göttingen, Germany; Boise S. Lukes’s Children’s Hospital, Boise, ID and Stanford University Lucile Packard Children’s Hospital Division of Pediatric Cardiology, Palo Alto, CA, USA

**Editorial commenting on ‘Late arrhythmias following Fontan completion: insights from a Korean Multicentre Fontan Registry of over 1000 extracardiac conduit surgeries’, by J. Yoon *et al*., https://doi.org/10.1093/eurheartj/euag123.**

Fontan patients continue to represent one of the most challenging cohorts for paediatric and adult congenital cardiologists alike. The Fontan population continues to grow rapidly as the operation is applied more broadly and patient survival continues to improve. Surgical advances, intentional surveillance, and expert arrhythmia management are among the factors leading to an estimated 30-year survival of over 80%, double that from 20 years ago.^1^ Indeed, it is no longer uncommon to care for a 40-year-old Fontan patient in the adult congenital heart disease (ACHD) clinic. Specialized ACHD centres now employ multidisciplinary approaches to comprehensive Fontan care, enlisting support from cardiologists, imaging specialists, hepatologists and electrophysiologists. A 2022 model estimated 47 881 persons were alive with a Fontan across 11 countries (US, Europe, Australia, New Zealand) with this number expected to reach nearly 60 000 by 2030.^2^ As we continue to fine tune the management of this population, national registries and databases can be mined to optimize clinical outcomes.

As Fontan patient survival improves, the burden of arrhythmia will also increase. Atrial arrhythmia remains the most important morbidity in the population and the subject of the article in this issue of Europace.^3^ The most common arrhythmia is the nearly ubiquitous intra-atrial re-entrant tachycardia, but other supraventricular arrhythmia can develop, including focal atrial tachycardia, atrioventricular node-dependent tachycardias, the rare twin AV reciprocating tachycardia, and as the population ages, atrial fibrillation. The burden of arrhythmia is determined not only by the underlying congenital defect but by the variant of Fontan surgery and perhaps most importantly, the age of the population in question. It is clear that the atrial arrhythmia burden has decreased since we abandoned the original atriopulmonary (AP) Fontan, but data comparing extracardiac conduit (ECC) and lateral tunnel (LT) Fontan remain conflicted. The answer depends on what you consider important and the lens through which you see these patients.^4–6^

Published in this issue of Europace are compelling data from a Korean nationwide registry.^3^ This multi-centre study analyzed outcomes in 1492 Fontan patients with a median follow-up of 14 years. Arrhythmias occurred in 13.3% (tachyarrhythmias 9.9%, bradyarrhythmias 6.8%) at a median age of 17 years, values that are remarkably similar to the 2014 New Zealand and Australian data from d’Udekem.^6^ Similarly, the AP and lateral tunnel (LT) Fontan increased arrhythmia risk when compared with the more modern ECC modification.^6^ Arrhythmias occurred in 53% of patients with AP Fontan, 31% with LT Fontan, and only 6% with ECC Fontan. Those who developed arrhythmias were older at their Fontan operation and were followed longer after Fontan completion. This is broadly consistent with the known late onset of arrhythmia in this population.^1^ Since decades separated the implementation of the different surgical approaches, these outcomes may be inherently challenging to compare. The AP Fontan was first performed in 1971,^7^ the LT Fontan in 1988^8^ and the ECC (although first introduced in 1990)^9^ was not widely embraced until the early to mid-2000’s. This era effect is critical as the first 10 to 15 post-operative years are typically relatively quiescent from an arrhythmia perspective.^4^ Although time-dependent analyses can be utilized (as in the present study) to mitigate disparities in duration of observation, the disparity in follow-up still limits the comparison of very late-onset arrhythmia estimates.^3^ Perhaps time will be the equalizer and as the ECC patients mature we will see their burden of arrhythmias approach those of the previous surgical iterations. The Korean data published here are no exception with follow-up of 26.6 years in the AP Fontan vs. a mere 11.2 years in ECC patients.^3^ The present study does, however, reflect a modern Fontan cohort with 80% having undergone ECC Fontan; and of these, favourable outcomes at 20 years (freedom from any arrhythmia 82.5%, tachyarrhythmia 86.9%, and bradyarrhythmia 90.5%).

Even if time proves that the ECC Fontan patients are less likely to develop arrhythmia, will these same arrhythmias become more challenging to treat? Prior iterations of the Fontan operation (AP and LT variants) typically permit direct transvenous atrial pacing for sinus node dysfunction and relatively facile catheter-based access for arrhythmias, either directly or by low-risk intracardiac transbaffle punctures.^10^ Conversely, by bypassing the atrial chambers entirely, the ECC Fontan adds layers of complexity to arrhythmia management. For instance, atrial pacing may require complex transpulmonary puncture procedures to reach viable atrial myocardium.^11^ Similarly, access to the atrial chambers for catheter ablation, usually requires either a technically challenging conduit or vena-caval puncture (or remote magnetic navigation at select centres).^12,13^ Such procedures may carry a risk for extracardiac haemorrhage and are only performed at expert centres. Thus, although the ECC Fontan may be associated with a reduced propensity for future arrhythmia,^3,14^ this operation may paradoxically (and substantially) increase the difficulty associated with its treatment.

The present findings should compel paediatric cardiologists and cardiothoracic surgeons to develop strategies to reduce the arrhythmogenic footprint of the operation. At least 3 key areas of focus may help address future arrhythmia associated with the ECC Fontan operations: (i) arrhythmia prevention, (ii) surgical modifications aimed at facilitating arrhythmia treatment, (iii) and a leverage of newer technologies. For instance, research into the root cause of sinus node dysfunction (e.g. by understanding the role of autonomics, fibrosis, genetics, and coronary vascular supply to the conduction tissues) may prevent the need for future pacing. Similarly, previously suggested radio-opaque ‘trapdoor’ patches may allow for easier access to the atrial chambers when pursuing future catheter ablation.^15^ Finally, new leadless pacing and energy sources may provide superior therapeutic options for the future cohort of ECC Fontan patients with arrhythmia. Such approaches may ensure that the lesser arrhythmia burden associated with the ECC Fontan need not paradoxically translate into a new set of challenges.

## Data Availability

The authors report that are no conflicts of interest.
